# Comparative genomics of *Cylindrospermopsis raciborskii* strains with differential toxicities

**DOI:** 10.1186/1471-2164-15-83

**Published:** 2014-01-29

**Authors:** Rati Sinha, Leanne A Pearson, Timothy W Davis, Julia Muenchhoff, Ryanbi Pratama, Aaron Jex, Michele A Burford, Brett A Neilan

**Affiliations:** 1 School of Biotechnology and Bimolecular Sciences, University of New South Wales, 2052 Sydney, NSW, Australia; 2 Australian Rivers Institute, Griffith University, 4111 Nathan, Queensland, Australia; 3 Faculty of Veterinary Science, University of Melbourne, Melbourne, Victoria, Australia

**Keywords:** *Cylindrospermopsis raciborskii*, Cylindrospermopsin, Cyanobacteria, Comparative genomics

## Abstract

**Background:**

*Cylindrospermopsis raciborskii* is an invasive filamentous freshwater cyanobacterium, some strains of which produce toxins. Sporadic toxicity may be the result of gene deletion events, the horizontal transfer of toxin biosynthesis gene clusters, or other genomic variables, yet the evolutionary drivers for cyanotoxin production remain a mystery. Through examining the genomes of toxic and non-toxic strains of *C. raciborskii*, we hoped to gain a better understanding of the degree of similarity between these strains of common geographical origin, and what the primary differences between these strains might be. Additionally, we hoped to ascertain why some cyanobacteria possess the cylindrospermopsin biosynthesis (*cyr*) gene cluster and produce toxin, while others do not. It has been hypothesised that toxicity or lack thereof might confer a selective advantage to cyanobacteria under certain environmental conditions.

**Results:**

In order to examine the fundamental differences between toxic and non-toxic *C. raciborskii* strains, we sequenced the genomes of two closely related isolates, CS-506 (CYN^+^) and CS-509 (CYN^-^) sourced from different lakes in tropical Queensland, Australia. These genomes were then compared to a third (reference) genome from *C. raciborskii* CS-505 (CYN^+^). Genome sizes were similar across all three strains and their G + C contents were almost identical. At least 2,767 genes were shared among all three strains, including the taxonomically important *rpoc1, ssuRNA, lsuRNA, cpcA, cpcB, nifB and nifH,* which exhibited 99.8-100% nucleotide identity. Strains CS-506 and CS-509 contained at least 176 and 101 strain-specific (or non-homologous) genes, respectively, most of which were associated with DNA repair and modification, nutrient uptake and transport, or adaptive measures such as osmoregulation. However, the only significant genetic difference observed between the two strains was the presence or absence of the cylindrospermopsin biosynthesis gene cluster. Interestingly, we also identified a cryptic secondary metabolite gene cluster in strain CS-509 (CYN^-^) and a second cryptic cluster common to CS-509 and the reference strain, CS-505 (CYN^+^).

**Conclusions:**

Our results confirm that the most important factor contributing to toxicity in *C. raciborskii* is the presence or absence of the *cyr* gene cluster. We did not identify any other distally encoded genes or gene clusters that correlate with CYN production. The fact that the additional genomic differences between toxic and non-toxic strains were primarily associated with stress and adaptation genes suggests that CYN production may be linked to these physiological processes.

## Background

Cyanobacteria are photosynthetic prokaryotes that thrive in a wide variety of habitats. Their occurrence in aquatic environments is of particular interest due to their ability to form dense and potentially toxic blooms under certain environmental conditions [1,2]. Some of the toxins produced include cyclic hepatotoxic peptides such as microcystin and nodularin [3], alkaloids such as the cytotoxic cylindrospermopsin (CYN) [4] and the neurotoxic saxitoxin (STX) [5], and organophosphates, such as anatoxin-a(s) [6]. These cyanotoxins, produced by over 40 species from 20 genera of cyanobacteria [7], have adverse health effects on humans and animals, and are a public health and environmental concern [3].

The toxin CYN first came into recognition following the poisoning of 149 people on Palm Island in 1979 [8]. Since then several animal poisonings have been recorded, including cattle mortalities [9]. Interestingly, several distantly related cyanobacterial species produce CYN, including *Aphanizomenon ovalisporum*[10], *Raphidiopsis curvata*[11], *Oscillatoria* sp. PCC 6506 [12]*, Anabaena lapponica*[13], *Lyngbya wollei*[14], *Umezakia natans*[15], *Raphidiopsis mediterranea*[16], *Anabaena bergii*[17] and *Cylindrospermopsis raciborskii*[18].

*C. raciborskii*, a filamentous diazotrophic cyanobacterial species, is a known producer of both cylindrospermopsin and saxitoxin [18], with the type of toxin produced apparently linked, in part, to geographic distribution. Although STX-producing *C. raciborskii* strains have been reported in South America [19], based on the current understanding, the most broadly distributed toxigenic members of this species produce CYN [20], with CYN producers (CYN^+^) having been reported in Australia, Asia, and New Zealand [21]. Additionally, *C. raciborskii* strains containing some *cyr* and *sxt* (saxitoxin biosynthesis) genes have also been found in South America, although these strains were found to be non–toxic [22]. There is mounting evidence for the global emergence of *C. raciborskii*, with the species exhibiting an increasingly cosmopolitan distribution. *C. raciborskii* was initially identified in tropical climatic regions, however, reports of its occurrence in temperate zones have increased drastically in the last decade [20,23-25].

Although the production of CYN and STX by cyanobacteria is well characterized and relates primarily to the presence of toxin-specific biosynthetic gene clusters [26,27], the mechanism for acquisition/loss of toxin gene clusters between and among closely related strains of *C. raciborskii* or a variety of distantly related cyanobacteria species [28] is not understood. It is also not known, which, if any, genes associated with the *cyr* gene cluster might be lost without loss of toxigenicity. Although a representative genome has been published for a toxigenic strain of *C. raciborskii*[19] there has been no large-scale characterization of the genome of other members of the species, or, specifically between and among toxic and non-toxic strains. Such comparisons may provide insight into how toxin clusters are shared/inherited, whether the differences between being toxic or non-toxic strains relates to a loss (e.g. through gene loss or mutational change) or gain (e.g. through cluster acquisition) of function, and what additional differences may exist between the genomes of toxic and non-toxic *Cylindrospermopsis* strains.

In the present study we conducted genome-wide comparisons of two closely related, but toxicologically distinct strains, CS-506 (CYN^+^) and CS-509 (CYN^-^), of *C. raciborskii* isolated from the same geographical region of Queensland, Australia and compared these with the published genome of the CYN^+^*C. raciborskii* CS-505 [19]. A primary aim of this study was to elucidate the minimal set of genes required for CYN production and explore whether or not this genomic locus extends beyond the *cyr* cluster. We also hoped to ascertain if toxin production influences the overall physiology of CS-506, by examining the putative metabolic roles of strain-specific genes. Our results are discussed within the context of the evolution and ecophysiology of these closely related cyanobacterial strains.

## Methods

### Cyanobacterial strains and culturing

*C. raciborskii* strains CS-506 and CS-509 were originally isolated from the Queensland waterbodies Solomon Dam (8.7242°S 146.594°E) in 1996 and Lake Julius (20.1315°S 139.723°E) in 1995, respectively. Detailed toxin and morphotype analyses of strains CS-506 and CS-505 were conducted in a previous study in which they were referred to as ‘form 1’ and ‘form 2’, respectively [29]. For the present study, strains were obtained from the Australian National Algae Culture Collection (ANACC), CSIRO Marine and Atmospheric Research, Hobart, Tasmania. Non-axenic cultures of these strains were grown in 250 ml culture flasks in Jaworski medium (JM) [30] at 25°C, under a light intensity of 25–30 μmol photons m^-2^ s^-1^ with a 12 h light/dark cycle.

### DNA extraction and quality control

To harvest cells, dense 250 ml cultures were filtered onto 3 μm pore size nitrocellulose membranes and washed with 2 volumes of JM to reduce contaminating bacteria to undetectable levels by 16S PCR (see below). High molecular weight DNA was extracted as previously described [31]. Briefly, cells were lysed with lysozyme and treated with proteinase K. The resulting lysate was then treated with 20% sodium dodecyl sulphate and cetyl trimethylammonium bromide and extracted with phenol/chloroform. DNA was precipitated with 2 volumes of ethanol, and 0.1 volume of 3 M sodium acetate then washed twice with 70% ethanol. Each sample was incubated at 4°C for 12–24 h to allow RNA degradation to occur and DNA quality was assessed spectrophotometrically and by gel electrophoresis. Only high molecular weight pure DNA samples were used for sequencing. DNA samples with a 260/280 nm absorbance ratio of 1.8-2.0, and a 260/230 nm absorbance ratio of 1.8–2.0 were considered pure.

The DNA samples were further quality-checked by amplification of the 16S rRNA gene by PCR. Primers specifically targeting the cyanobacterial 16S rRNA gene (27FL/809R), and bacterial 16S rRNA gene (27FL/1494R) [32] were used to this end. PCR was performed using the following conditions: initial denaturation at 94°C for 2 min, followed by 30 cycles of denaturation at 94°C for 30 s, annealing at 55°C for 30 s, extension at 72°C for 1 min, then a final extension at 72°C for 30 s. The resulting amplicons were sequenced using the BigDye Terminator kit (Invitrogen) and analyzed using Bioedit [33]. DNA samples that yielded pure *C. raciborskii* 16S rRNA gene sequences were finally submitted for genome sequencing. These steps ensured that all *C. raciborskii* DNA samples used for genome sequencing were free from contaminating heterotrophic bacterial DNA.

### Genome sequencing and comparative analyses

Paired-end indexed libraries were prepared from purified DNA fragments of approximately 320 bp in length. Genome sequencing was performed using the Genome Analyzer IIx sequencing platform and TruSeq SBS v4 GA kit. The raw reads generated were 100 bp in length. Raw read quality was visualized using FastQC software (Babraham Bioinformatics) with default settings. All raw reads were filtered for quality (mean phred > 20) and end-trimmed (10 bp at 5' and 3') using custom Perl scripts. Paired-reads passing these quality filters were used to estimate the genome size for each strain using the program khmerfreq [34] using kmer = 17 and then assembled using SOAPdenovo software [34] with different kmer lengths (57–64), with the final assembly selected based on overall assembly size, number of contigs and contig size.

The optimal assemblies for *C. raciborskii* CS-506 and CS-509 were annotated using homology-based (Integrated Microbial Genomes (IMG) [35] and Rapid Annotations Subsystems Technology (RAST) [36]) and predictive modeling (Glimmer [37], Genemarks [38]) approaches. We used the published genome of the CYN-producing *C. raciborskii* strain CS-505 [19], also isolated from Solomon Dam, Queensland, Australia, as a reference genome for comparative assessment of these annotation methods. Glimmer 3 and Genemarks vastly overestimated gene number, when compared to the previously published reference genome for *C. raciborskii* strain CS-505. For ease of annotation, and to be consistent with the prediction of CS-505 genes, RAST was used for the final analysis. NRPS-PKS predictor [39] and antiSMASH [40] software were used to identify non-ribosomal peptide synthetase (NRPS) and polyketide synthase (PKS) domains. Additionally, further annotation of genes encoding hypothetical proteins was conducted using the program InterProScan [41] or the pfam database [42].

Following annotation of each genome, we conducted pair-wise comparisons of the gene-sets predicted for each strain using BLASTn [43]. Genes were considered common to two taxa in pair-wise comparisons if reciprocal BLAST hits had (a) an e-value ≤ 1*10^-5^, (b) ≥ 90% nucleotide identity and (c) a difference between query and alignment length of ≤ 20 bp. Genes not meeting these criteria in any pair-wise comparison were considered strain-specific. To ensure that genes 'missing' from each strain based on these BLASTn comparisons were not absent due to gaps in the assembly or misannotation, we mapped the filtered reads from both CS-506 and CS-509 to the published CS-505 sequence using the program SOAPAligner [44]. The bioinformatic prediction of these strain-specific genes was further tested using PCR for a subset of sequences (n = 13; Additional file 1). Briefly, primers targeting genes that were absent from only one of the three *C. raciborskii* strains, CS-505, CS-506 or CS-509, were designed using primer-BLAST (NCBI). PCR was performed using the following conditions: initial denaturation at 94°C for 2 min, followed by 30 cycles of denaturation at 94°C for 30 s, annealing at 59°C for 30 s, extension at 72°C for 1 min, and a final extension at 72°C for 30 s. Suitable positive and negative controls were used in all PCR experiments and approximately 20 ng of template DNA was used in each reaction. Amplicons were visualized using an agarose gel electrophoresis unit. 1% agarose in TAE gels were stained in ethidium bromide and subsequently viewed using a UV transilluminator. Amplification of an expected size PCR product was used to confirm the presence of a gene.

Structural and overall nucleotide variation between and among *C. raciborskii* CS-505, CS-506 and CS-509 genomes was assessed at the whole genomic level by comparative alignment using the '-nucmer' (−−maxmatch) and 'dnadiff' packages of the program Mummer 3 [45]. Specific nucleotide variation between and among protein-encoding genes and within non-coding regions was assessed by comparative alignment of each genome using the Smith-Waterman alignment algorithm of the program Burrows-Wheeler Aligner (BWA: [46]). Genes common to all three *C. raciborskii* strains were each aligned as orthologous clusters using Muscle [47] and the alignments assessed for synonymous, non-synonymous and indel mutations. To ensure only high-confidence single nucleotide polymorphisms (SNPs) were included in our analysis, we mapped the raw reads for each sample to its respective assembly (using Bowtie2) [48] and filtered the initial SNP calls using the Neighbor-Quality Scoring method [49] in which only SNPs covered to a depth ≥ 5 with reads having a phred mapping quality of ≥ 20 and flanked to the 5' and 3' by at least 3 bases with a phred mapping quality of ≥ 15 were retained for subsequent analysis. Read mapping quality was assessed using Samtools v 0.1.19 [50]. Using these SNP data, various population genetic metrics ( e.g. segregating sites, synonymous and non-synonymous SNPs per bp) were calculated using custom-perl scripts.

The data sets supporting the results presented in this manuscript are available in the following repository: Integrated Microbial Genomes [IMG] repository, unique persistent identifier 12992 and 12991 and NCBI short read archive under accession numbers: SRR1042336 and SRR1041118 for *C. raciborskii* strains CS-506 and CS-509, respectively.

## Results and discussion

### Genomic structure

The genomes of *C. raciborskii* strains CS-506 and CS-509 were sequenced via the bridge amplification method on an Illumina genome analyzer. Both genomes were sequenced to a depth of ~828 and 459 fold, respectively. The total assembly size for each genome was 4.1 Mb (N50 = 25,000 bp; total scaffolds 698) for strain CS-506 and 4.0 Mb (N50 = 56,411 bp; total scaffolds 319) for CS-509 (Table 1), which is comparable to that of the reference strain, CS-505 (3.9 Mb). The largest scaffolds assembled were 67,497 bp for CS-506 and 188,708 bp for CS-509. Overall, the draft assemblies for strains CS-506 and CS-509 yielded 103 and 65 scaffolds >10 kb, 8 and 19 scaffolds >40 kb, and 0 and 5 scaffolds over 100 kb, respectively. The G + C content of both genomes was similar (approximately 40.9%) and comparable to that of the reference strain (40.2%), as well as other filamentous cyanobacterial genomes i.e. *Raphidiopsis* sp. [28] and *Anabaena* sp. presently available in the public databases (40–41.5%) (NCBI). In the absence of a physical map for these genomes, structural variation among the assemblies was interpreted with some caution. Nonetheless, based on pair-wise comparisons, CS-505 (CYN^+^) and CS-509 (CYN^-^) show a higher level of structural synteny as compared to CS-506 (CYN^+^) (Additional file 2). Overall the CS-509 assembly could be aligned in syntenic blocks with >95% of the CS-505 assembly, whereas CS-506 showed alignment synteny with only 93.6% of this genome. The CS-506 assembly, relative to the CS-505 genome, also contained a larger number of breakpoint (8,315), translocation (1,111), insertion (2,846) and tandem insertion (9) events than the pair-wise alignment of CS-509 and CS-505, further evidence of the greater similarity between the CS-505 and CS-509 genomes, compared to the similarity between the toxic strains CS-505 and CS-506. The CS-509 assembly, in comparison contained 8,036 breakpoint, 927 translocation, 2,539 insertion and 5 tandem insertion events.

**Table 1:** Genome assembly statistics of the three strains

**Genome characteristics**	***C. raciborskii *** **strain**		
	**CS-506**	**CS-509**	**CS-505**
Size (Mb)	4.1	4.0	3.9
G + C content (%)	41.1	40.7	40.8
N50 (bp)	25,000	56,411	NA
N90 (bp)	412	268	NA
Largest scaffold (bp)	67,497	1,88,708	2,59,000
Scaffolds > 10 kb	103	65	NA
Scaffolds > 40 kb	8	19	NA
Scaffolds > 100 kb	0	5	NA
CDS*	3,268	3,416	3,452
Unique CDS*	176	101	181
Common CDS*	2,767	2,767	2,767
rRNA genes*	8	6	9
tRNA genes*	44	40	42
Genes with functional annotations (%)	36	34	55

N50, largest contig size, and other information relevant to the assembly of the genomes. NA = Data not available, CDS = coding sequence, *Total number.

CS-505 and CS-509, contrary to their different toxic phenotypes, were also significantly more similar in sequence than CS-505 and CS-506, with 8,200 SNPs (72.8% in coding regions) and 13,405 SNPs (75.8% in coding regions) observed between the genomes of these pairs respectively. Based on multiple pair-wise alignment of the three *C. raciborskii* genomes, we established an orthologous relationship among all three strains for 2,917 of the 3,418 protein coding genes annotated for CS-505 [19]. Comparative alignment of these assemblies revealed 99.5% nucleotide identity (representing 3,599,169 alignable bases) among all three strains (Additional file 3). For these orthologous genes, we detected 9,460 (3.3 per kb) and 4,766 (1.8 per kb) SNPs between CS-505 and CS-506 and between CS-505 and CS-509, respectively, relating to a mean nt identity of 99.6 and 99.8%, respectively. Notably, among the genes found to be identical among all three strains were *rpoc1* (RNA polymerase subunit), *cpcA* and *cpcB* (phycocyanin alpha and beta subunits) and *nifB* and *nifH* (nitrogen fixation proteins), all of which are widely utilized for phylogenetic analyses of cyanobacteria. In a separate analysis, we also noted 99.8% sequence identity among *ssuRNA* and *lsuRNA* (small and large subunits of ribosomal RNA). Hence, although these loci have proven useful for the molecular classification of cyanobacteria to the species level, our results suggest that they are not useful for differentiating *C. raciborskii* strains. Some of the more variable genes under low mutational selective pressure based on the accumulation of coding (i.e., non-synonymous) to non-coding (i.e., synonymous) SNPs for both CS-506 and CS-509 that may be worthy of further exploration as intra-specific markers for *Cylindrospermopsis* are a putative Random Associated Mysterious Protein (RAMP) superfamily protein, CRC_01868 (3.7 and 4.1% nt variability for CS-506 and CS-509, respectively), a hemolysin A homolog, CRC_02719 (3.4 and 4.9% variability) and a HAD-superfamily hydrolase, CRC_00377 (3.4 and 2.1% variability).

Interestingly, a large proportion of the differences between CS-506 and CS-509 in the shared, orthologous genes related to non-synonymous (NS) mutations, with these being more than twice as common in the former (n = 5,971 SNPs) than the latter (n = 2,419) relative to CS-505. By contrast, synonymous (S) SNPs in these orthologous genes were relatively equal in number between the two strains (2,495 and 2,054 for CS-506 and CS-509, respectively). The higher rate of NS SNPs in these genes between CS-506 and CS-505 in comparison to CS-509 and CS-505 may be suggestive of significant differences in the ecological niches of or selective pressures on CS-505 and CS-506 despite their shared toxic phenotype and the close proximity of their geographic origin.

**Figure 1:**
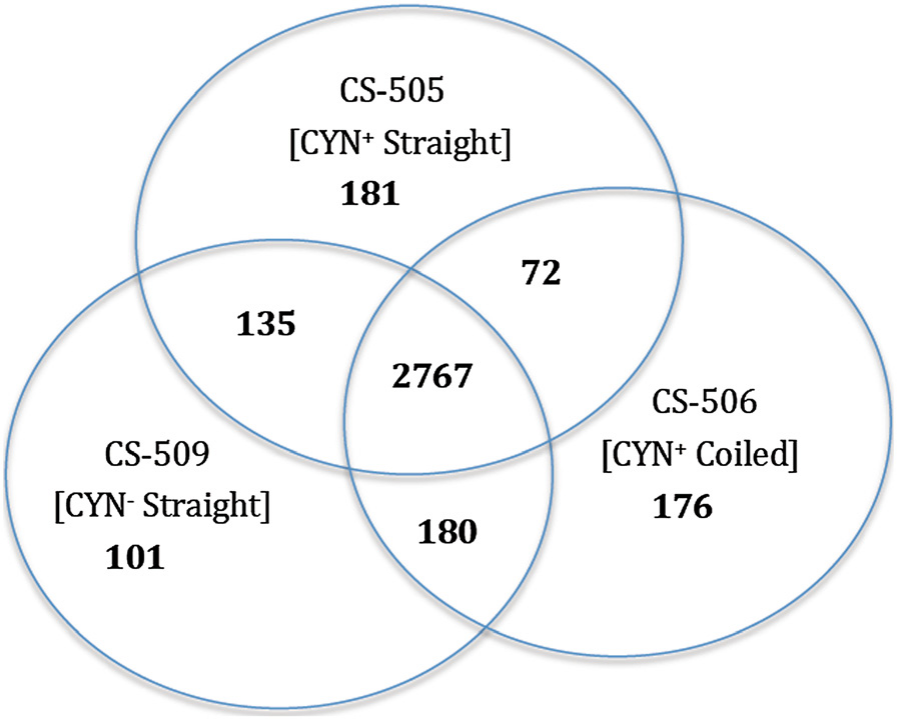
**Number of exclusive and shared genes among *C. raciborskii *strains CS-505, CS-506 and CS-509. Major phenotypic differences, including toxicity (CYN**^**+/−**^**) and morphology (straight/coiled) are also indicated.** 297, 73 and 233 genes could not be confidently designated as common or strain-specific and were therefore excluded from our study.

**Figure 2:**
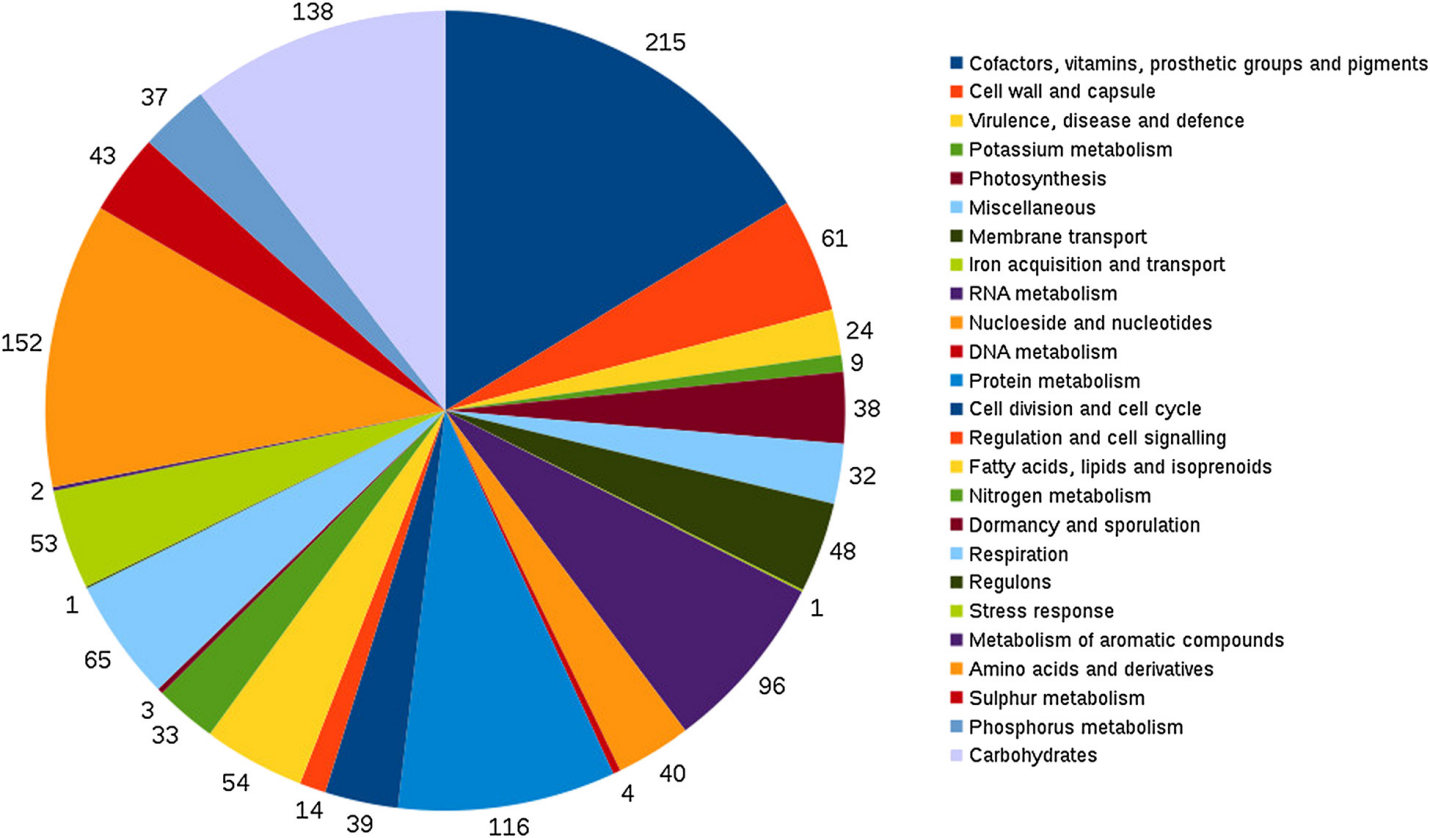
**Classification of functionally annotated common genes between strains CS-505, CS-506 and CS-509.** Fifty-eight percent of genes in this “core” genome are hypothetical and are not represented in the figure.

In addition to these alignment based comparisons, we independently assessed the gene composition and putatively identified functional differences between the three strains using RAST [51]. This approach also allowed identification of novel genes encoded in either CS-506 or CS-509 but not found in CS-505. Based on RAST prediction, the CS-506 and CS-509 assemblies were predicted to encode 3,268 and 3,416 protein encoding genes, respectively (see Table 1), compared to the 3,452 currently annotated for CS-505 [19]. With RAST identifying 2,767 of these CDS to be shared as homologs among all three *C. raciborskii* strains, CS-506, CS-509 and CS-505 (Figures 1 and 2), representing 82%, 84.8% and 81.8% of all predicted CDS, respectively.

### Conservation among *C. raciborskii* predicted metabolomes

The genes shared between/among all three *C. raciborskii* strains included those associated with key metabolic pathways, such as photosynthesis, nitrogen and phosphorus metabolism. Many of these pathways appear to be highly conserved across the three *C. raciborskii* strains and are also similar to primary metabolic pathways identified in other species of cyanobacteria [52-54]. Although all core genes required for photosynthesis in cyanobacteria [52] were present in each *C. raciborskii* strain, these genes were distributed among eight distinct operons (e.g. *psaAB, psbCD*, *petCA*, *petBD, atpIHGFDAC, coxBAC, chlDHI* and *chlNBL*). This arrangement of photosynthesis genes as eight separate operons is also found in other cyanobacteria, including *Nostoc punctiforme* ATCC 29133 [53], *Raphidiopsis curvata*, and *Raphidiopsis mediterranea* but distinct from related, non-cyanobacterial species, such as *Rhodobacter sphaeroides*, which organize their photosynthesis genes into a single operon [55]. It is possible that one long continuous photosynthesis gene cluster is ancestral in the bacteria, with subsequent genomic re-arrangement into separate operons occurring more recently in some taxa.

Nitrogen metabolism genes, including those for nitrogen fixation, ammonium, nitrate and nitrite assimilation and heterocyst development were also conserved among CS-505, CS-506, and CS-509. These genes clustered into several distinct operons, including those for nitrogenase (*nifB, fdxN, nifS, nifU, nifH, nifD, nifK, ORF, nifE, nifN, nifX, ORF, ORF, nifW, hesA, hesB, fdxH*), and nitrite/nitrate uptake and reduction (*nirA, nrtA, nrtB, nrtC, nrtD, narB*). Such operons have been described in other cyanobacteria, including *N. punctiforme* ATCC 29133*, Anabaena* PCC 7120, *A. variabilis* ATCC 29413 [56], and *A. variabilis* PCC 7120 [57].

We also observed the presence of an identical set of phosphorus metabolism genes, displaying high synteny in all three strains. These comprise the *pho* regulon (*phoU, phoR, phoB, pstA, pstB, pstC* and *pstS*), inorganic phosphatase, transhydrogenases (subunits alpha and beta) and alkaline phosphatases, and are required for phosphorus metabolism [54]. The genes *pstA, pstC* and *pstB* form one cluster while the histidine kinase *phoR* and the transcriptional regulator *phoB* form another. A similar arrangement of phosphorus metabolism genes has been observed in the genomes of *Microcystis aeruginosa* PCC 7806 [58] and *Raphidiopsis mediterranea.*

### Substantial differences in transporters among *C. raciborskii* strains

ABC transporters facilitate the translocation of ions or macromolecules across biological membranes, including the export of substances toxic to the cell [59]. Relative to CS-506 and CS-509, CS-505 was enriched for genes involved in transport, export, and nutrient uptake. The CS-505 genome encodes for a large number of ATP Binding Cassette (ABC) transport-related genes, comprising 4.1% of its total genome. In contrast, ABC-transporter genes comprised only 2.3 and 2.4% of the total genes in CS-506 and CS-509, respectively. The enrichment of ABC transporters in CS-505 was mainly limited to those responsible for the transport of glycerophospholipids [34,60], with 24 of these genes present exclusively within this strain. The strain-specific nature of these specialized ABC transporters suggests a specific ecological adaptation that is not explicitly linked to CYN production, but may relate to membrane structure and permeability. The number of ABC transporters in other cyanobacteria was found to be significantly lower, for example in *Nostoc punctiforme* ATCC 29133, ABC transporters comprise only 0.03% of the genome [53]. Similarly, RAST analyses revealed that in *M. aeruginosa* ABC transporters comprise a mere 0.002% of the genome [51]. Other transport-related genes identified in our study strains include amino acid, N-acetylglucosamine related, energy-coupling bacterial, and tripartite ATP-independent periplasmic (TRAP) transporters, all of which were found in consistent, but low, numbers (<1%).

### Strain-specific genes

**Figure 3:**
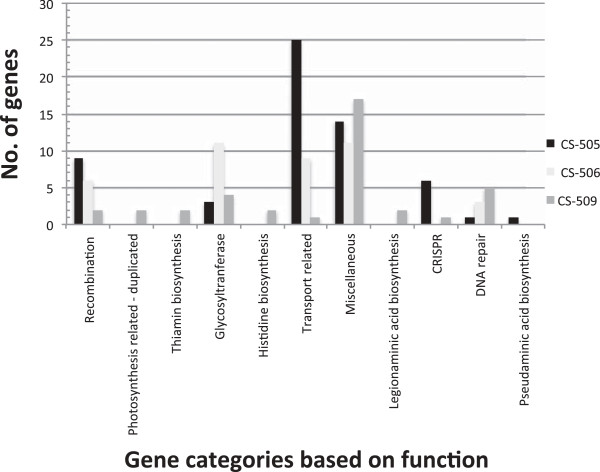
**Classification of strain-specific genes in the study strains possibly associated with ecophysiological adaptations.** Miscellaneous category represents peptidases, proteases, methyltransferases, and and folate, thiamin and cell wall biosynthesis.

Five percent of all genes in CS-506 were specific to this toxic strain (although 4.1% of these were annotated as hypothetical proteins). Similarly, 2.9% and 5.2% of all genes in CS-509 (CYN^-^) and CS-505 (CYN^+^), respectively, were strain-specific (1.7% and 3.5% of these were hypothetical). The strain-specific genes identified seemed to be largely associated with environmental response and adaptation, particularly for phage counteraction, recombination, DNA repair, transport and nutrient uptake, and stress (Figure 3). Interestingly, although a similar number of DNA repair genes were present in all the three strains (approximately 0.9 -1% of their genomes), the three gene sets involved in this process were not identical. Approximately 15% of the total DNA repair related genes in CS-509 were strain-specific, whereas 7.2% of all DNA repair-related genes in strains CS-505 and CS-506 were unique to those strains. Many of these genes were found in multiple loci. The genome of the CYN-producing strain CS-505 was also enriched with interspaced short palindromic repeats (CRISPR) related genes, which comprised 0.6% of its total genome. CRISPR systems are defence machineries used by bacteria and archaea against bacteriophage [61]. In comparison, CS-506 and CS-509 contained between 0.3-0.46% CRISPR related genes in their genomes. Strain CS-505 also contained an additional set of *cas1,2* genes containing a CRISPR locus, not present in its counterparts.

The presence of strain-specific genes and proteins has been observed in other cyanobacterial genomes and proteomes. For example, a comparative proteomic analysis of six toxic and non-toxic *Microcystis aeruginosa* strains reported a large diversity in the protein expression profiles of each strain, with a significant proportion of the identified proteins appearing to be strain-specific. The study found that strains of *M. aeruginosa* species differ in adaptation-related processes, rather than metabolic ones. Additionally, no protein produced exclusively by toxic or non-toxic strains was found, including the Mcy proteins responsible for microcystin biosynthesis [62]. The observed proteome diversity led to the conclusion that *M. aeruginosa* strains are ecotypes adapted to survival in a particular environmental niche rather than phylogenetically distinct subgroups. Likewise, our data suggests that strains CS-505, CS-506 and CS-509 are ecotypes adapted to specific ecological niches that exist within the same broader geographic location.

### Toxic strain-specific genes

**Figure 4:**
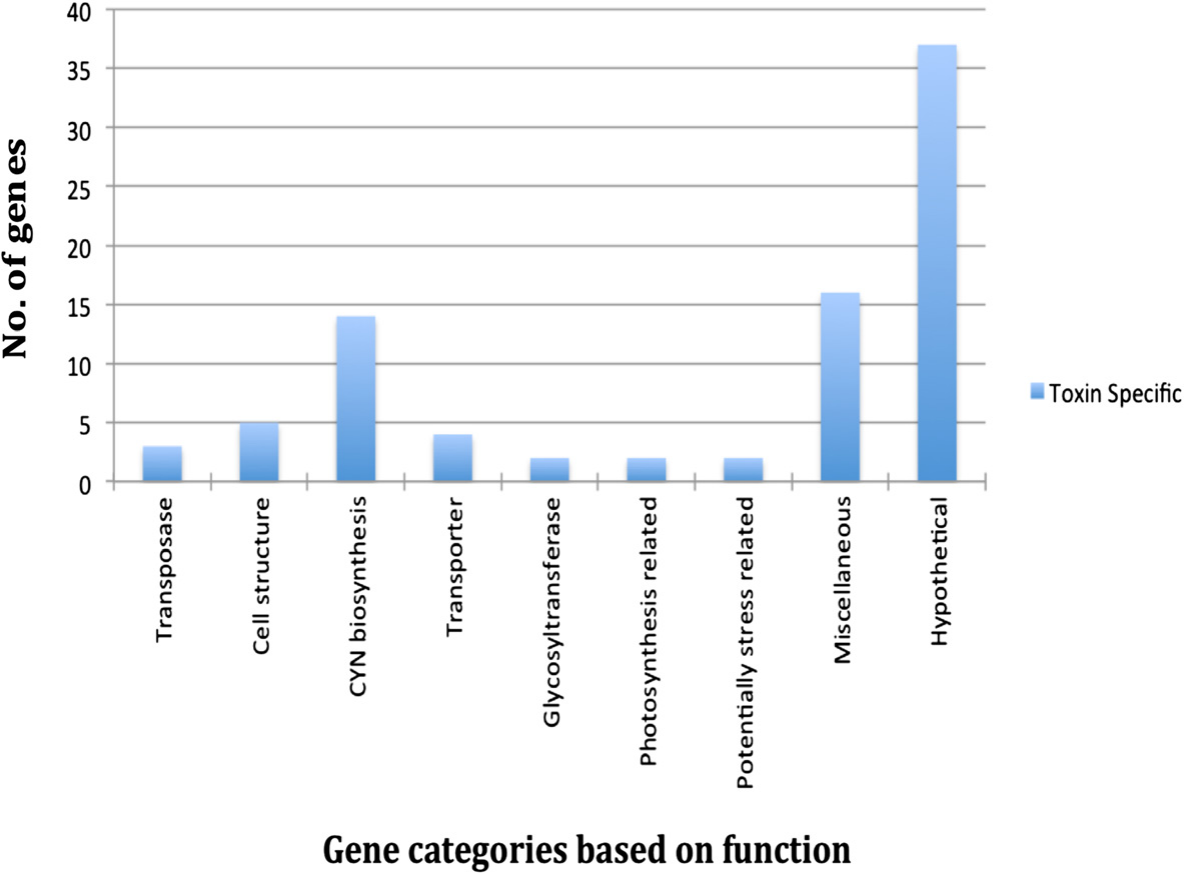
**Classification of toxic strain (CS-505 and CS-506)-specific genes.** The miscellaneous category represents genes responsible for protein processing, sialic acid metabolism and cell division.

We identified 72 genes (Figure 4) common to the CYN-producing strains (CS-505 and CS-506), but absent from the non-toxic strain (CS-509). Of these, 34 were annotated as hypothetical. The *cyr* gene cluster (which has already been elucidated in strains AWT205 [26] and CS-505 [19]) was identified in CS-506, but not CS-509. The *cyr* gene cluster encompasses 43 kb and encodes 15 ORFs. It comprises genes responsible for the complex biosynthesis of the CYN, namely an amidinotransferase (*cyrA*)*,* a NRPS/PKS hybrid gene (*cyrB*)*,* four PKS genes (*cyrC, cyrD, cyrE* and *cyrF*), amidohydrolases (*cyrG* and *cyrH*), as well as genes for tailoring reactions (*cyrI, cyrJ,* and *cyrN*), putative transport (*cyrK*), and regulation (*cyrO*). It also contains two transposase genes (*cyrM* and *cyrL*[26]), indicating the potential for the horizontal transfer of toxicity.

**Figure 5:**
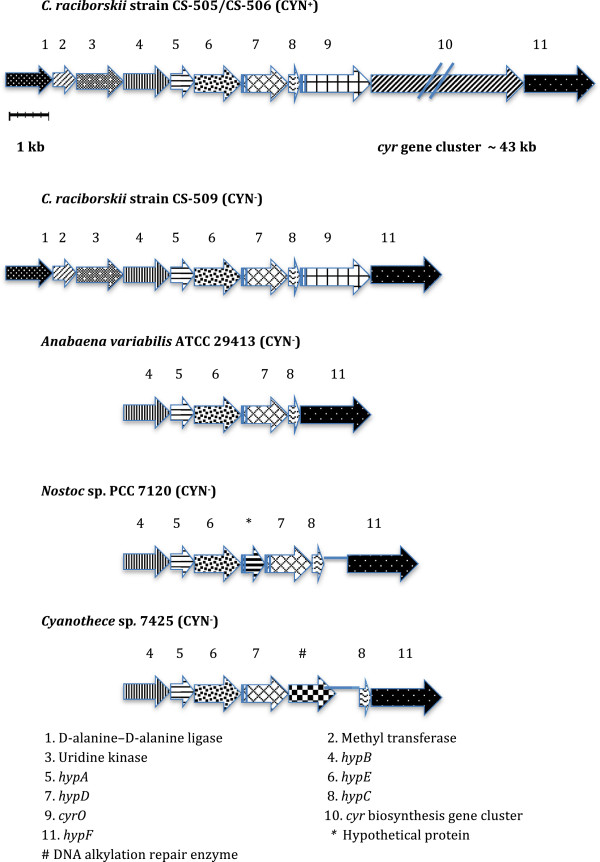
**Arrangement of genes present in the *cyr *cluster neighbourhood or equivalent genomic location in *C. raciborskii *strains CS-505 and CS-509, *Anabaena variabilis *ATCC 29413*, Nostoc punctiforme *PCC 7120 and *Cyanothece *sp. 7425.** This figure highlights the absence of the *cyr* gene cluster in the non-CYN producing *C. raciborskii* strain. Note due to space limitations, the *cyr* cluster has been truncated in this figure**.**

Although the genes comprising the *cyr* cluster appear largely conserved among CYN-producing cyanobacteria [26,63,64], their location and arrangement differs between/among genera [63]. The *cyr* cluster in *C. raciborskii* CS-506 is flanked by *hyp* (hydrogenase pleitrophy) genes (Figure 5), which play a critical role in the maturation of hydrogenases (i.e., NiFe metalloenzymes) [65] and cluster with 20 other genes, which together are responsible for the expression of the active iron metalloenzyme. This appears to be typical for CYN-producing *Cylindrospermopsis* strains (e.g. AWT205 [26] and CS-505 [19]). In contrast, the *cyr* gene cluster in *Aphanizomenon* sp*.* is flanked by a transposase and a putative transcriptional attenuator gene [64]. The differential genomic location of the *cyr* cluster in various CYN-producing species suggests that the cluster may be a mobile genetic element capable of jumping between and within cyanobacterial genomes. In further support of this hypothesis, the G + C contents of the *cyr* gene clusters in CS-505 and CS-506 (44-49%, except *cyrI* 37%) were much higher than the G + C contents of the flanking genes (38-40%) or of the overall genome (40–40.2%). This finding is consistent with previous studies which showed that the G + C content of the *cyr* gene cluster in *Aphanizomenon* sp*.* 10E6 was >44%, and significantly higher than for neighboring genes [66].

Although the *cyr* cluster was missing from CS-509, a single *cyr* gene, *cyrO* was present between the *hyp* genes. The precise function of CyrO remains to be determined as does its relationship to the *cyr* cluster. CyrO has low homology to WD repeat proteins, which have diverse regulatory signal transduction roles as well as to ATPases associated with diverse activities (AAA) family proteins which participate in chaperone like functions such as the assembly, operation and disassembly of protein complexes [26]. The G + C content of *cyrO* was found to be 43, consistent with the *cyr* gene cluster, and higher than the flanking genes, suggesting that it may have been acquired by horizontal gene transfer or has moved from a high GC region of the genome. In contrast to its location in the *C. raciborskii* genome*, cyrO* in *Raphidiopsis curvata* D9 is separate from and distally located to the *cyr* gene cluster [63]. Likewise, no clear orthologs of this gene were found in CYN-producing *Aphanizomenon* sp. 10E6 [28]. The absence of *cyrO* from the *cyr* gene cluster or genomes of other CYN-producing species, suggests that this putative CYN regulatory protein may have an alternative function, or at least is not essential for CYN-production.

Although the presence of the complete *cyr* gene cluster was the most obvious toxic strain-specific trait identified in this study, a few other genes were common to CS-505 and CS-506, but absent from CS-509. These included genes putatively involved in transport and protein processing. The production of cyanotoxins is energetically expensive to the cell therefore the expression of additional ABC transporters may facilitate the uptake of nutrients required by toxic strains. Single genes responsible for cell division, cell wall capsule biosynthesis, and DNA repair were also found. Another gene, whose function in cyanobacteria has not yet been defined, was also found to be specific to the toxic strains. This gene was homologous to vanadium dependent bromopeptidases (VBPOs), which play a role in the hydrogen peroxide-dependent oxidization of halides in eukaryotes [67]. VBPOs can also act as antioxidants, removing hydrogen peroxide, a byproduct of photosynthesis detrimental to cells. In cyanobacteria, VBPOs are thought to be associated with organic compounds that infer allelopathic attributes [67] and therefore selective advantages to toxic strains.

### Potential mechanism for transfer, acquisition and/or loss of the *cyr* cluster

A recent study comparing over twenty strains of non-toxic, STX and CYN-producing *C. raciborskii*, found no correlation between phylogeny and toxicity [28]. Stucken et al. 2010 suggested that the absence of toxicity in some strains of *C. raciborskii* was due to the absence or loss of the *cyr* cluster, rather than to point mutations or partial deletions [28]. Our results support this hypothesis and suggest that *cyr* genes were acquired by horizontal transfer in CS-505 and CS-506, and lost *in toto* from the CS-509 genome.

Evidence of acquired toxicity via horizontal gene transfer (HGT) and its subsequent loss has been documented for other species of cyanobacteria. For example a recent study [68] hypothesized that saxitoxin production was either gained independently via HGT in STX-producing strains, or that it was gained by a common ancestor, and lost after several generations from non-STX producers. Genomic analysis of STX-producing *Anabaena circinalis* ACBU02 and a non-STX-producing ACFR02 showed that the latter strain contained four of the 26 *sxt* biosynthetic pathway genes, advocating the occurrence of genetic deletion. Another study examining the loss of microcystin production in *Plankothrix* species found that certain non-toxic strains had lost up to 90% of the *mcy* gene cluster [69]. These strains, however, still contained the genes flanking the *mcy* gene cluster along with remnants of the transposable regions.

### Presence of novel metabolite clusters

**Figure 6:**
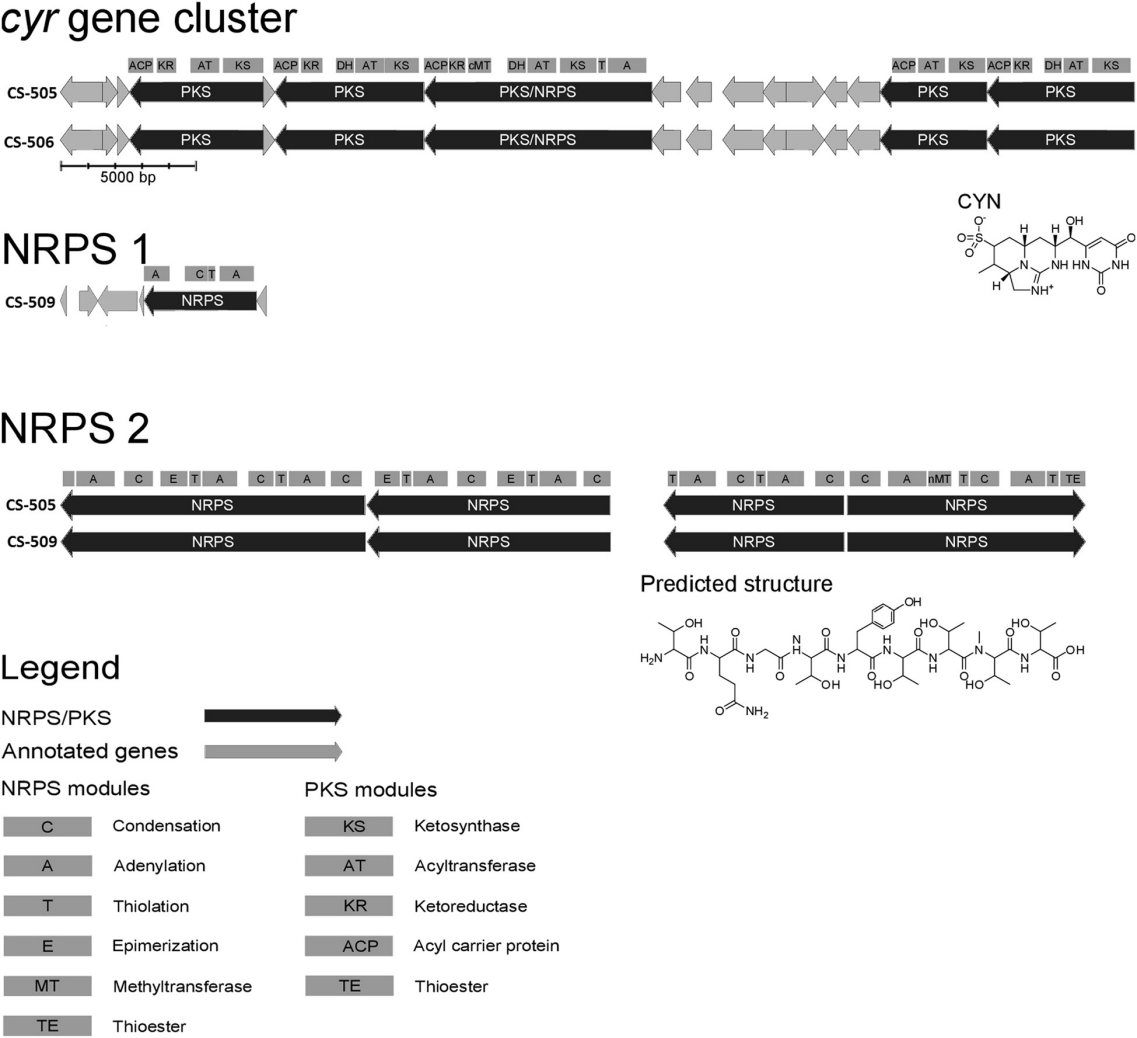
**Secondary metabolite clusters identified in the CS-505, CS-506 and CS-509 genomes.** The structure prediction was performed using the AntiSMASH program [40], utilising the monomer prediction of NRPSPredictor2 [39].

Two additional secondary metabolite clusters were detected in the *C. raciborskii* genomes examined in this study. These clusters, designated NRPS1 and NRPS2, are functionally uncharacterized at this stage (Figure 6). Cyanobacteria are producers of an array of bioactive secondary metabolites [70]. Such compounds are often produced by non-ribosomal peptide synthetases (NRPS), and polyketides synthases (PKS) and are of interest due to their toxic or therapeutic properties, including antimicrobial, antifungal, or antitumor properties [70].

The cryptic NRPS1 gene cluster identified in the non-toxic *C. raciborskii* strain CS-509 (ORF 2370–2375) is 7,000 bp long and encompasses 6 ORFs, including a 4,209 bp NRPS, a hypothetical protein (ORF 2373), a major family facilitator (*MFS_1* gene, ORF 2372), and a sterol desaturase (ORF 2374). An incomplete NRPS1 cluster was also identified in the reference strain CS-505. However, in this cluster, the NRPS gene exists as a truncated (600 bp) fragment and the *MFS_1* gene is missing. Sequence-based analyses suggest that the NRPS and *MFS_1* genes in the CS-509 NRPS1 cluster were acquired via HGT from a common ancestor of *Anabaena variabilis* ATCC 29413 and *Cyanothece* sp., which possess genes with 82% and 86% similarity to NRPS and *MFS_1*, respectively. The peptide sequence of NRPS1 is also 92% similar to an NRPS in *Raphidiopsis brookii* D9, the function of which is unknown. The *R. brookii* D9 NRPS, however, possesses an additional thiolation domain, putatively required for the production of a dipeptide.

The second cryptic cluster identified in this study, NRPS2 occurs in the straight morphotypes, CS-505 and CS-509, but not in the coiled strain CS-506. NRPS2 is approximately 25 kb in length and comprises 8 ORFs (numbered 2680–2687 in CS-505 and 727–734 in CS-509), including a probable hydroxylase and an acyl carrier protein reductase. This cluster is present in the same location in both CS-505 and CS-509 and is flanked by identical genes in both genomes. AntiSMASH analysis suggests that the NRPS2 cluster is involved in the biosynthesis of an octapeptide, which could possibly be the unidentified *C. raciborskii* toxin reported by Falconer et al. 1999 [71]. However, further experimentation, including mutagenesis of the biosynthesis genes or heterologous expression of the complete cluster, combined with chemo-analytical studies are required to verify this.

### Genetic divergence and plasticity

We observed numerous instances of genome duplication in the three study strains, often in multiloci positions. These ranged from photosynthesis-related genes in CS-509 to a unique transposase gene, present as ORFs 10 and 487 in CS-506. Further, two identical gene clusters, each comprising five genes encoding hypothetical proteins, which bear >20% nucleotide similarity to kinases and a gene encoding a DUF324 protein of unknown function, were also found in the CS-509 genome. The genes that constitute these twin gene clusters also show a high degree of similarity (92%) to a cluster of hypothetical proteins in *Cyanothece* sp. PCC 7424.

Nasvall et al. 2012 proposed and subsequently validated the innovation-amplification-divergence (IAD) model, based on a study conducted on over 3,000 generations of the bacterium *Salmonella enterica*[47]*.* This theory proffers that genes initially amplify to a higher copy number, following which, one of the extra copies suffer mutations. This eventually leads to divergence of the strain from its co-strains. It is possible that the same phenomenon is in play here, and the different strains of *C. raciborskii* are gradually diverging through gene duplication, mutation and deletion events.

Further, the highly plastic nature of the *C. raciborskii* genomes is highlighted by the presence of transposase genes, which are found in close proximity to genes with G + C contents highly deviant from the average G + C content of the *C. raciborskii* genomes. Additionally, G + C rich genes were found amidst genes of lower G + C content. This along with the presence of strain-specific genes and integrases reiterates the polymorphic nature of the *C. raciborskii* genome. Numerous other instances, such as the varying G + C content of the *cyr* gene cluster compared to the flanking *hyp* gene cluster and the presence of transposase genes adjacent to CRISPR arrays, advocate construction of these genomes via HGT events.

Other cyanobacteria also display evidence of genome plasticity. For example 6.8% of the *Microcystis aeruginosa* PCC 7806 genome encodes putative transposases as well as a large number of atypical genes not found in other cyanobacteria [58]. Similarly, a large proportion of the *Nostoc punctiforme* ATCC 29133 genome encodes unique proteins (29%), insertion sites and multilocus repeat sequences [53].

### Genes associated with morphological variation between CS-505, CS-506 and CS-509

In addition to differing in toxin-production, CS-505, CS-506 and CS-509 differ in physical morphology; CS-505 and CS-509 are straight, while CS-506 has a coiled trichome [29]. The differing morphologies may be associated with different survival strategies. For example coiled CS-506 have been observed to dominate over straight CS-505 in environmental blooms, likely in relation to the preference among predators, such as *Daphnia*, for straight, rather than coiled morphotypes [29]. To better understand the genetics underpinning cell morphology in this species, we examined the three *C. raciborskii* genomes for the presence of morphotype-specific genes. Around 200 genes were common to the straight morphotypes (CS-505, CS-509) but absent from the coiled strain (CS-506). As expected, numerous genes related to cell wall and capsule biosynthesis were exclusive to the straight morphotypes. These include genes responsible for the biosynthesis of capsular and extracellular polysaccharide and murein hydrolases, which play a role in the regulation of cell wall growth in bacteria [32]. Additionally, while the *Mre* gene that is responsible for cell shape in bacteria was found in all three strains, we observed a total of five SNPs in the gene sequences, three of which were common to the straight morphotypes. The inactivation of the *Mre* gene in *Anabaena* sp. PCC 7120 has previously been shown to convert the rod-like shape of the cell to a spherical form [61]. While, further experimentation is required to validate whether the *Mre* genes in the coiled *C. raciborskii* strains are active or not, or whether the presence of the SNPs in the straight morphotypes affects the translation of these genes in some way, it is possible that this gene plays a role in *C. raciborskii* morphology. We also found several genes exclusive to the straight morphotypes, many of which are putatively involved in cell division, transport, DNA repair, recombination and stress response. Whether or not these genes play a role in cell morphology remains to be determined.

## Conclusions

Our results suggest that CS-505, CS-506 and CS-509 represent distinct ecotypes of *C. raciborskii,* with subtle genetic differences resulting from the niche selective pressures of their specific but geographically similar environments.

This is the first example of genome comparison between closely related toxic and non-toxic *C. raciborskii* strains. As expected, the genomes of these strains were very similar. However, subtle genomic differences alluding to the adaptability of the species were identified. Moreover, numerous examples of strain-specific genes, genes with disparate G + C contents, duplicated and/or rearranged genes, as well as transposases and integrases were observed. Taken together, these results underpin the plasticity of the *C. raciborskii* genome and its potential to evolve in the face of selective pressures. This ability to adapt may help explain the recent invasion of *C. raciborskii* from tropical to temperate climates around the globe.

Most significantly, we demonstrated that toxicity in this species is dependent on possession of the *cyr* gene cluster, as no other candidate secondary metabolite gene clusters were positively correlated with toxicity. While previous attempts to mutate and thus confirm the role of *cyr* genes have been unsuccessful, we can now conclude with a high degree of certainty that the *cyr* cluster is in fact responsible for CYN biosynthesis. Additionally, the non-toxic CS-509 strain lacked the complete suite of *cyr* genes, but possessed a unique cryptic secondary metabolite gene cluster, NRPS1, the function of which is unknown.

The description of two closely related, but toxigenically different *C. raciborskii* genomes can be considered a starting point for further molecular studies into the regulation of CYN production and its native role in this species. For example, transcriptomic and proteomic analyses examining the effects of chemophysical parameters, such as light, nutrients, and trace elements, on toxic and non-toxic strains can provide insight into the environmental drivers for toxin production. Since the occurrence of this potentially toxic cyanobacterium is on the rise due to increased eutrophication and global warming [24,72], it is imperative to gain a better understanding of the various aspects of its physiology and the integral role played by CYN production. This will further facilitate better prediction and management of harmful cyanobacterial blooms.

## Competing interests

The authors declare that they have no competing interests.

## Authors’ contributions

RS carried out the molecular genetics experiments, the bioinformatics analyses, genome assembly and annotation and genome comparisons, as well as the drafting of the manuscript. AJ supervised all the bioinformatics analyses and performed SNP analyses and multiple alignments/comparisons between genomes. RP participated in the NRPS/PKS cluster detection and analyses. BN and MB designed the project and helped in its co-ordination. LP, TD and JM participated in the analyses and interpretation of results and helped draft the manuscript. All authors read and approved the final manuscript.

## Supplementary Material

Additional file 1 **Primers used to confirm the results of genome sequencing and bioinformatic analyses.** Where genome sequencing and bioinformatic analyses were inconclusive, PCR screening was used to verify the presence or absence of genes. Appropriate negative and positive controls were used for all PCR reactions. + indicates target gene present. - indicates target gene was absent. Click here for file

Additional file 2 **Structural and overall nucleotide variation between *C. raciborskii *** **CS-505, CS-506 and CS-509.** Genomes were assessed at the whole genomic level by comparative alignment using the '-nucmer' (−−maxmatch) and 'dnadiff' packages of the program Mummer 3. Click here for file

Additional file 3 **a: SNP data comparisons of CS-506 and CS-509 relative to CS-505. b: SNP data comparisons of CS-506 and CS-509 relative to CS-505;** SegSi = segregating sites, variable positions which in this case are equivalent to total SNPs; Syn = synonymous; NonSyn = non-synonymous; Stop = mutations which appear to have introduced a premature stop codon in a gene; Ambig = ambiguous mutations that usually occur where the reference has a non-standard IUPAC nucleotide code (R, Y, etc.). Click here for file
