# Supplementary material for: Comparative genomics of Cylindrospermopsis raciborskii strains with differential toxicities
Source: BMC Genomics. 2014 Jan 29;15:83. doi: 10.1186/1471-2164-15-83 (PMC3922686; doi:10.1186/1471-2164-15-83)
Supplement: Additional file 1 — Primers used to confirm the results of genome sequencing and bioinformatic analyses. Where genome sequencing and bioinformatic analyses were inconclusive, PCR screening was used to verify the presence or absence of genes. Appropriate negative and positive controls were used for all PCR reactions. + indicates target gene present. - indicates target gene was absent. [file 1471-2164-15-83-S1.zip › 5246470181107225_add1.pdf]

Primer name	Target gene	CS505	CS506	CS509	Amplicon size	Primer sequence
2721F	Glycosyl transferase, group 1	+	-	-	802	GAGTGCATGGCGCTAATGAGGT
2721R	Glycosyl transferase, group 1	+	-	-	802	TGACATGGCTTCTAGTGGTGGCA
2722F	Methyltransferase FkbM family	+	-	-	694	ACGCACCAGTTTTCGCTAATGAAGT
2722R	Methyltransferase FkbM family	+	-	-	694	TCAGCCACACTGTCTCCTGCCA
800F	Putative serine protease	+	-	-	884	TGGCACACAGGCGATCGCAAA
800R	Putative serine protease	+	-	-	884	GCGATCGCTGAAGTTGCTGGA
351F	CRISPR-associated RAMP Cmr2	+	-	-	928	TCCGGCTGACTGGTGGGTGT
351R	CRISPR-associated RAMP Cmr2	+	-	-	928	CCCCCAAGCCCATTGTTCCCA
352F	CRISPR-associated RAMP Cmr3	+	-	-	874	CCGGAGAAGGTGCTTGGGCT
352R	CRISPR-associated RAMP Cmr3	+	-	-	874	TGTGCCAGTCCAGGGGTGAGT
647105335F	Peptidase S8 and S53, subtilisin, kexin, sedolisin	+	-	-	991	GGCACAGCAAACCCGGGACA
647105335R	Peptidase S8 and S53, subtilisin, kexin, sedolisin	+	-	-	991	CGGTTAGGGCGCTGGTGGTG
647105837F	Hypothetical protein	+	-	-	453	CTCAAGTCGTATGGCAAACAAAGCG
647105837R	Hypothetical protein	+	-	-	453	TGTTCGTTACTTCTCCTGTCCGGCT
647106111F	Cell envelope-associated LytR-CpsA-Psr transcriptional attenuators	+	-	-	712	GCATCGGGCTCTTTCGCACG
