# Supplementary material for: Comparative genomics of Cylindrospermopsis raciborskii strains with differential toxicities
Source: BMC Genomics. 2014 Jan 29;15:83. doi: 10.1186/1471-2164-15-83 (PMC3922686; doi:10.1186/1471-2164-15-83)
Supplement: Additional file 1 — Primers used to confirm the results of genome sequencing and bioinformatic analyses. Where genome sequencing and bioinformatic analyses were inconclusive, PCR screening was used to verify the presence or absence of genes. Appropriate negative and positive controls were used for all PCR reactions. + indicates target gene present. - indicates target gene was absent. [file 1471-2164-15-83-S1.zip › 5246470181107225_add2.pdf]

647106111R	Cell envelope-associated LytR-CpsA-Psr transcriptional attenuators	+	-	-	712	TGTTGGCCATTCCTGTGCGAGT
647106112F	Putative ATP-dependent protease	+	-	-	766	GCCCCCTTTCCTCCACCGGA
647106112R	Putative ATP-dependent protease	+	-	-	766	GTGCAAAAGCAGTGCGGAGGG
647107190F	CRISPR-associated protein Cas1	+	-	-	500	TCCGAAGAAGGTCGTACAGTTGCG
647107190R	CRISPR-associated protein Cas1	+	-	-	500	TTGGCACGTCCATAACGCTCACC
647107615F	Hypothetical protein	+	+	-	535	GATGCTCGAGCCGCCTGGG
647107615R	Hypothetical protein	+	+	-	535	CCTCTTGGGGGCCCACCTCC
647107647F	N-linked Glycosylation in Bacteria	+	+	-	989	TGCGGGTGGGTCAATAGGTGC
647107647R	N-linked glycosylation in bacteria	+	-	-	989	TTCCTTGACGCCCTGCTGCC
647107590F	Restriction-modification system,	+	-	-	614	TGGGGTTGAGTGGTTGGGGGA
647107590R	Restriction-modification system,	+	-	-	614	TGGGGACCTCCTCGCGAACG
