# Supplementary material for: Comparative genomics of Cylindrospermopsis raciborskii strains with differential toxicities
Source: BMC Genomics. 2014 Jan 29;15:83. doi: 10.1186/1471-2164-15-83 (PMC3922686; doi:10.1186/1471-2164-15-83)
Supplement: Additional file 2 — Structural and overall nucleotide variation between C. raciborskii CS-505, CS-506 and CS-509. Genomes were assessed at the whole genomic level by comparative alignment using the '-nucmer' (−−maxmatch) and 'dnadiff' packages of the program Mummer 3. [file 1471-2164-15-83-S2.docx]

|  |  | CS505 vs. CS509 | | CS505 vs. CS506 | |
| --- | --- | --- | --- | --- | --- |
| Sequences |  | [REF] | [QRY] | [REF] | [QRY] |
|  | TotalSeqs | 93 | 2517 | 93 | 3050 |
|  | AlignedSeqs | 93 | 1367 | 92 | 1480 |
|  |  | -100.00% | -54.31% | -98.92% | -48.52% |
|  | UnalignedSeqs | 0 | 1150 | 1 | 1570 |
|  |  | 0.00% | -45.69% | -1.08% | -51.48% |
| Bases | Total Bases | 3879030 | 4034670 | 3879030 | 4183928 |
|  | Aligned Bases | | 3698640 |  | 3631332 |
|  |  |  | -95.35% |  | -93.61% |
|  | Unaligned Bases | | 180390 |  | 247698 |
|  |  |  | -4.65% |  | -6.39% |
| Feature Estimates | Breakpoints | 8036 | 2367 | 8315 | 2372 |
|  | Relocations | 12 | 22 | 9 | 21 |
|  | Translocations | 927 | 77 | 1111 | 91 |
|  | Inversions | 0 | 1 | 0 | 1 |
|  | Insertions | 2539 | 1193 | 2846 | 1043 |
|  | Insertion Sum | 523389 | 311431 | 594538 | 262072 |
|  | Insertion Avg | 206.14 | 261.05 | 208.9 | 251.27 |
