# Supplementary material for: Comparative genomics of Cylindrospermopsis raciborskii strains with differential toxicities
Source: BMC Genomics. 2014 Jan 29;15:83. doi: 10.1186/1471-2164-15-83 (PMC3922686; doi:10.1186/1471-2164-15-83)
Supplement: Additional file 3 — a: SNP data comparisons of CS-506 and CS-509 relative to CS-505. b: SNP data comparisons of CS-506 and CS-509 relative to CS-505; SegSi = segregating sites, variable positions which in this case are equivalent to total SNPs; Syn = synonymous; NonSyn = non-synonymous; Stop = mutations which appear to have introduced a premature stop codon in a gene; Ambig = ambiguous mutations that usually occur where the reference has a non-standard IUPAC nucleotide code (R, Y, etc.). [file 1471-2164-15-83-S3.docx]

|  | Comparison of CS506 and CS509 relative to CS505 | | | |  |
| --- | --- | --- | --- | --- | --- |
| Total number segregating sites for whole genome | 19154 |  |  |  |  |
| Total number segregating sites for coding regions | 14302 |  |  |  |  |
|  |  |  |  |  |  |
| Total number segregating sites for noncoding regions | 4852 |  |  |  |  |
| Total number SNPs for whole genome | 21603 |  |  |  |  |
| Total number SNPs for coding regions | 16128 |  |  |  |  |
| Total number SNPs for noncoding regions | 5475 |  |  |  |  |

| Strain | Coding | SegSi | Syn | NonSyn | Stop | Ambig |
| --- | --- | --- | --- | --- | --- | --- |
| CS506 | 3233398 | 10143 | 2671 | 6391 | 204 | 542 |
| CS509 | 3233398 | 5970 | 2459 | 3137 | 37 | 299 |
|  |  |  |  |  |  |  |
